# A compiled dataset of ready-mix concrete Environmental Product Declarations for life cycle assessment

**DOI:** 10.1016/j.dib.2023.109852

**Published:** 2023-11-26

**Authors:** Jonathan Michael Broyles, Juan Pablo Gevaudan, Nathan Collin Brown

**Affiliations:** a The Pennsylvania State University, Department of Architectural Engineering, 104 Engineering Unit A, University Park, PA, 16802, USA; b The Pennsylvania State University, Department of Material Science and Engineering, PA, USA

**Keywords:** Sustainability, Embodied carbon, LCA Midpoints, Cradle-to-gate LCA, Concrete mixtures, Ready-mix concrete plants, Ready-mix concrete companies, Ready-mix plant locations

## Abstract

The carbon footprint of a concrete structure is directly affected by the selected concrete mixture proportions. To better understand the influence of different concrete mixtures, data was collected from Environmental Product Declarations (EPDs). Data from 39,213 U.S.A. ready-mix concrete EPDs was obtained from public repositories provided by the American Society for Testing and Materials and the National Ready Mixed Concrete Association. The EPDs in .pdf format were analyzed using a custom Python script to extract useful information for building designers, sustainability practitioners, and researchers including: life cycle assessment (LCA) midpoints (Global Warming Potential, Ozone Depletion Potential, Acidification Potential, Photochemical Ozone Creation Potential, Abiotic Depletion, Total Waste Disposed, and Consumption of Freshwater), concrete strength classes, declared unit, concrete curing time, production components, concrete manufacturers’ company and plant locations, and additional LCA information. Both the dataset and an example of the Python script used to extract the information from the EPDs are provided. This dataset enables users to quickly assess the environmental impacts (including the Global Warming Potential) of different concrete mixtures without the need for extensive data collection and analysis. In summary, this dataset provides environmental information about concrete mixtures to aid civil engineering and architectural researchers, sustainability consultants, building engineering practitioners, and environmental policymakers to make sustainability-informed decisions when specifying concrete in the U.S.A.

Specifications Table

**Table:** 

Subjects	Sustainability and the Environment, Civil and Structural Engineering, Architecture
Specific subject area	Ready-mix concrete A1-A3 LCA midpoints, including the Global Warming Potential, and other information extracted from EPDs, such as the concrete strength and ready-mix plant location.
Data format	Analyzed
Type of data	.csv/.xlsx file (the ready-mix concrete EPD dataset with labels, numerical, and categorical data). .py file (a snippet of Python code to extract information from an EPD).
Data collection	The data recorded in the .csv file was extracted from 39,213 EPDs using a custom Python script (see the .py file). Reported LCA midpoints (e.g., Global Warming Potential and Ozone Depletion) were recorded for each concrete mixture and were subdivided by LCA stage (A1-A3, A1, A2, A3). Additionally, the strength of the concrete, the declared unit, product description, and LCI information were collected. The ready-mix concrete producer's company, plant, and plant locations were also extracted from the EPDs. Lastly, the internet links to access the EPDs are provided.
Data source location	The database contains information extracted from EPDs provided online by the American Society for Testing and Materials[1] and the National Ready Mixed Concrete Association[2] made available during the period of February 2023 to April 2023. All EPDs (and corresponding ready-mix concrete plants) are based on concrete mixtures across the United States of America.
Data accessibility	Repository name: Mendeley Data [3] Data identification number: 10.17632/r4jgxk2mhn.2 Direct URL to data: https://data.mendeley.com/datasets/r4jgxk2mhn/2

## 1 Value of the Data

• The concrete EPD dataset allows building designers and sustainability practitioners to easily compare concrete mixtures from various ready-mix concrete plants for the primary LCA midpoints to support sustainable design decisions in the built environment. The LCA midpoints include Global Warming Potential, Ozone Depletion Potential, Acidification Potential, Eutrophication Potential, Photochemical Ozone Creation Potential, Abiotic Depletion (non-fossil fuel and fossil fuel), Total Waste Disposed (non-hazardous and hazardous), and consumption of freshwater. The LCA midpoints are provided for each individual A1, A2, and A3 LCA stage, in addition to the summation of the A1-A3 LCA stages.
• The dataset contains additional information extracted from the ready-mix concrete EPDs including the mixture description, concrete compressive strength, declared unit, product components, the Life Cycle Inventory products and sources, and the street locations of the ready-mix concrete plants. The locations of the ready-mix concrete producers, including each ready-mix concrete plant, are provided to help consultants identify concrete mixtures that are near their project site.
• This dataset can be reused to further understand the environmental effects of the production of different concrete mixtures, which benefits building sustainability consultants, building practitioners, architects, civil and structural engineers, concrete plant manufacturers, structural concrete institutes (e.g., the American Concrete Institute, Structural Engineering Institute) and policymakers. Statistical analyses can be conducted to provide current baseline values of LCA midpoints for concrete mixtures of various compressive strength classes. Lastly, ready-mix concrete manufacturers can compare nationally the environmental footprint of their concrete mixtures to other ready-mix concrete manufacturers, encouraging the development of more sustainable concrete production.

## 2 Data Description

This dataset compiles ready-mix concrete manufacturer, concrete mixture, and environmental information from 39,213 Environmental Product Declarations (EPDs) of various concrete mixtures currently available in the U.S.A. The ready-mix concrete EPDs were downloaded from public repositories provided by ASTM [1] and NRMCA [2], with a custom Python script (see the accompanying .py file) employed to extract key information from the EPDs. The EPDs were followed according to ISO 14040 [4], ISO 14044 [5], and ISO 21930 [6]. Table 1 outlines the information obtained from the EPDs and presented in the dataset, including the ready-mix concrete manufacturer, ready-mix concrete plant location, general EPD information, engineering information regarding the concrete mixture proportions, LCA midpoints, and the Life Cycle Inventory (LCI) variability, and sources of LCI data as reported in the EPDs.

**Table 1:** The column headers in the EPD Dataset, grouped by category.

Category	Column header
Concrete Manufacturer Information	Concrete Company
	Company Location (Street, City, State, Zip)
	Concrete Plant
	Plant Location (Street, City, State, Zip)
General EPD Information	EPD Program Operator
	EPD Date of Issue
	EPD Valid Until Date
Concrete Information	Mix Label
	Mix Description
	Concrete Compressive Strength
	Concrete Curing Time
	Declared Unit
	Product Components
Life Cycle Assessment Midpoints	Global Warming Potential (A1-A3, A1, A2, A3) in kg CO_2_-eq
	Ozone Depletion Potential (A1-A3, A1, A2, A3) in kg CFC-11-eq
	Acidification Potential (A1-A3, A1, A2, A3) in kg SO_2_-eq
	Eutrophication Potential (A1-A3, A1, A2, A3) in kg N-eq
	Photochemical Ozone Creation Potential (A1-A3, A1, A2, A3) in kg O_3_-eq
	Abiotic Depletion, Non-fossil (A1-A3, A1, A2, A3) in kg Sb-eq
	Abiotic Depletion, Fossil (A1-A3, A1, A2, A3) in MJ
	Total Waste Disposed, Hazardous & Non-hazardous (A1-A3, A1, A2, A3) in kg
	Consumption of Freshwater (A1-A3, A1, A2, A3) in m^3^
Life Cycle Inventory Variability	Percent Mixing Truck Energy
	LCI Manufacturing Variability
	LCI Cement Accounts for Percent Energy
	LCI Cement Impact Variation
Life Cycle Inventory Data Sources	Admixture (accelerating)
	Admixture (air-entraining)
	Admixture (hardening accelerator)
	Admixture (other)
	Admixture (plasticizing)
	Admixture (retarding)
	Admixture (superplasticizing)
	Admixture (waterproofing)
	Aggregate (crushed)
	Aggregate (lightweight)
	Aggregate (natural)
	Aggregate (other)
	Barge Transport
	Carbon Cure
	Cleaning Chemicals
	Diesel
	Electricity
	Fly Ash
	Municipal Water
	Natural Gas
	Non-Hazardous Solid Waste
	Oils, Lubricants and Greases
	Portland Cement
	Portland Limestone Cement
	Propane
	Rail Transport
	Ship Transport
	Silica Fume
	Slag Cement
	Truck Transport
	Other
Internet Link / Source to Access the EPD	EPD Source Link

**Fig. 1:**
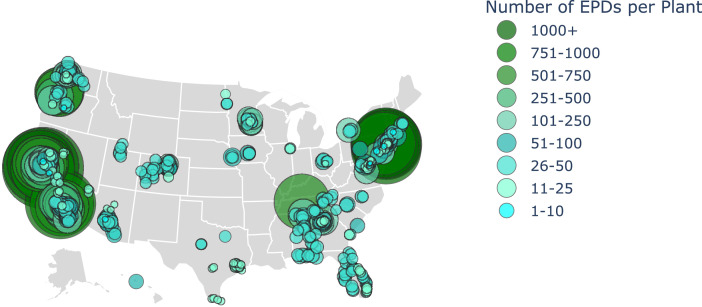
Distribution of ready-mix concrete plants with EPDs across the U.S.A. in the EPD dataset.

A key aim of this dataset is to provide high fidelity information of the environmental impacts for current concrete mixtures. Existing baseline reports such as the CLF 2021 [7] and NRMCA 2022 [8] reports exclude useful information, such as the complete list of product components in a concrete mixture and the primary sources of LCI data. Additionally, this dataset represents a broad distribution of ready-mix concrete EPDs currently available across the U.S.A. Ready-mix concrete EPDs from 37 different ready-mix concrete manufacturers and 389 unique ready-mix concrete plants were collected, as detailed in Table 2. Fig. 1 shows the geographic distribution of EPDs.

**Table 2:** Concrete manufacturers and concrete plants (alphabetized).

Ready-mix concrete manufacturer	Ready-mix concrete plants
Altaview Concrete	American Fork Plant, Logan Plant, North Salt Lake Plant, West Haven Plant, West Jordan Plant
Argos	Armour Drive Plant, Atlanta Division Plant, Doraville Plant, Glenwood Plant, Smyrna Plant
AVR Inc. & Affiliates	Apple Valley Plant, Buffalo Plant, Burnsville Plant, Elk River Plant, Ham Lake Plant, Maple Grove Plant, Monticello Plant, South St. Paul Plant
Bayview Redi Mix Inc.	Bayview Redi Mix Plant
BURNCO Colorado	Denver Plant, Jeffco Plant, Windsor Plant
Cadman / Heidelberg Materials	Bellevue Plant, Everett-Smith Plant, Ferndale Plant, Foster Road Plant, Issaquah Plant, Orchards Plant, Port of Portland Plant, Redmond Plant, Seattle Plant, Sky River Plant, Woodinville Plant
CEMEX	19th Avenue Plant, 34th Avenue Plant, 7th Street Plant, Alabaster-Pelham Plant, Alpharetta-North Fulton Plant, Antioch Plant, Apache Junction Plant, Apex Plant, Arcola Rail Yard Plant, Baymeadows Plant, Baytown Plant, Berkeley Plant, Big Bend Plant, Bradenton Plant, Brownsville Plant, Buckeye Plant, Buford (RMUSA) Plant, Bunnell North Plant, Camp Verde Plant, Cantonment (Dual) Plant, Carroll Canyon Plant, Carson City Plant, Cemco 10 Moorsville Plant, Central South Plant, Chattanooga Jersey Plant, Chattanooga River Plant, Cocoa Plant, College Park Plant, Columbia Plant, Compton Plant, Concord Plant, Coolidge Plant, Corona RM Plant, Cullman Plant, Cutten Road Plant, Dalton Plant, Daphne Plant, Davenport RK Plant, Delray Plant, Demopolis Plant, Dothan Plant, Douglasville Plant, Downtown-Marietta Plant, Downtown SA RM Plant, East Orlando Plant, Edinburg Plant, Ehren Cut-Off Plant, Ellington Plant, Enterprise Plant, Fairfield Portable Plant, Farmersville Plant, Flagstaff Plant, Florence-Industrial Plant, Fontana RM Plant, Fort Myers Plant, French Camp Plant, Friant Plant, Ft. Pierce Plant, Ft. Walton Plant, Gainesville RK Plant, Galveston Plant, Greeneville Plant, Harlingen Plant, Harvest-Highway 53-Lafarge Plant, Higley Plant, Hockley Plant, Hollywood Plant, Holmes RM (JV) Plant, Humble RM Plant, Huntsville (Dual) Plant, Inglewood Plant, Intel Plant, Irondale Plant, Irvine Plant, Johns Creek Plant, Katy RM (JV) Plant, Kingsport Plant, Kyle Canyon Plant, Lake Park Plant, Lakeshore (Dual) (Lafarge) Plant, Lemoore Plant, Lincoln Plant, Lithonia Plant, Lockhart RK Plant, Los Angeles Plant, Los Banos Plant, Losee Plant, Lytle Creek RM Plant, Maricopa Plant, Marietta-Owenby Drive Plant, Marysville Plant, Maynardville Plant, McDuffie Rd. Plant, Melbourne RK Plant, Merced Plant, Midtown-Armour Plant, Mid-Town Miami Plant, Mission Plant, Mission Valley Plant, Missouri City RM (JV) Plant, Modesto Plant, Montgomery-Metro Plant, Montgomery-Wares Plant, Morgan's Point (La Porte) Plant, Morristown Plant, Mt. Belvieu Plant, Navarre Plant, Navigation RM Plant, Neyland Drive Plant, Niceville (Villa Tasso) Plant, North Miami Plant, North Vero Plant, Oakland Plant, Ocala Plant, Oildale Plant, Old River Plant, Opelika Plant, Orange Plant, Orlando Plant, Oxnard RM Plant, Palomar Escondido Plant, Panama City-Main Plant, Perkins Plant, Perris RM Plant, Pier 92 Amador Plant, Pima Plant, Pleasanton Plant, Pompano Plant, Portable Rexcon 1 Plant, Prescott Plant, Prospect Avenue Plant, Redlands RM (Dual) Plant, Regency Park Plant, Reno Plant, Rockford Plant, Rosenberg Plant, Roseville Plant, Rothwell Plant, San Carlos Plant, San Jose Plant, San Juan Capistrano Plant, San Tan Plant, Santa Barbara Plant, Santa Clara Plant, Santa Puala RM Plant, Sarival Plant, Schertz RM Plant, Seagrove Plant, Sloan Plant, South Fresno Plant, South Lauderdale Plant, South Miami Plant, Spring Plant, St. Augustine Plant, St. Petersburg Plant, Stafford Plant, State Rd. 80 (WP8 FL) RM Plant, Stuart Plant, Sun City Plant, Sunrise Plant, Tomball Plant, Tracy Plant, Tric Plant, Trinity Plant, Troy Plant, TSMC Plant, Tuscaloosa (Dual) Plant, Union City Plant, Valkaria Plant, Webster Plant, West Palm Beach FL Readymix Plant, West Plant, West Sacramento Terminal Plant, Wiggings Pass (North Naples) Plant, Woodstock Plant
Central Concrete	Hayward Plant, Martinez Plant, Oakland Plant, Pleasanton (wet) Plant, Queens Lane (dry) Plant, Queens Lane (wet) Plant, Redwood City Plant, Redwood City B Plant, San Francisco Plant, South San Francisco (wet) Plant, Stockton (wet) Plant
Centre Concrete Co.	State College Plant
Corliss Resources, Inc.	Enumclaw Plant, Federal Way Plant, Puyallup Plant, Sumner Plant
CWC-WSG	Baker Flats Plant, Ephrata Plant, Othello Plant, Quincy Plant, Wenatchee Plant
Eastern Concrete	Bayonne Plant, Bogota Plant, Broadway Plant, Howell Plant, Newark Plant, North Bergen Plant, Roseland Plant, West Nyack Plant
Hawaiian Cement	Halawa Plant #1, #3
Holcim - Aggregate Industries	Bannock Plant, Belle Plaine Ready-Mix Plant, Beltsville Plant, Bladensburg Plant, Buffalo Ready-Mix – Hopkins Plant, Chantilly Plant, Costilla Plant, DFW RMX Plant, Empire Ready-Mix Plant, Everett Plant, Fargo Ready-Mix (dry) Plant, Fargo Ready-Mix (wet) Plant, Forest Lake Ready-mix Plant, Fort Totten (DC) Plant, Ft. Collins Plant, Grand Forks Ready-Mix Plant, Jessup Plant, Kirby Road Plant, Lancaster Ready-Mix – Genesee Plant, Manassas Plant, Maple Grove Ready-Mix Plant, Minneapolis Ready-Mix Plant, Newport Ready-Mix Plant, Portable LRT Ready-Mix Plant, Rexcon Plant 8, Rockville East Plant, Saugus Plant, Texas Plant, Tonawanda Ready-Mix River Rd. Plant, Waltham Plant
Hooker Creek Companies, LLC	Bend Plant, Madras Plant, Redmond Plant
Ingram Ready Mix	Pearland #35 LLC Plant
Knife River Corporation	Coffee Lake Plant, Hillsboro Plant, Linnton Plant, Prineville Plant, Sundial Plant
Liberty Ready Mix	Dixon 2 Plant, Dixon 3 Plant, Grimes 1 Plant
Martin Marietta - Smyrna Ready Mix Concrete, LLC.	Arvada Plant, Chambers Plant, Del Camino Plant, Quivas Plant, Rock Creek Plant, Valmont Plant
Nashville Ready Mix	Visco Dr. – Nashville Plant
National Ready Mix	Artesia Plant, Canoga Park Plant, Glendale Plant, Irvine Plant, Irwindale Plant, Moorpark Plant, Santa Clarita Plant, South Gate Plant, Sun Valley Plant, Van Nuys Plant, Vernon Plant
O&G Industries, Inc.	Bridgeport Plant, Danbury Plant, Harwinton Plant, Southbury Plant, Stamford Plant
Platte River Concrete	Chalco Plants 1–5, 7
Prairie Material, LLC	Yard 32 Plant, Yard 33 Plant
Ready Mix USA	Harvest Plant
Riverbend Materials	Corvallis Plant, Hilroy Plant, RBWest Plant, Wildish Plant
Robertson's Ready Mix	Anaheim Plant, Carroll Canyon Plant, El Cajon Plant, Gardena Plant, Mira Mar Plant, Otay Mesa Plant, Rialto Plant, Vernon Plant
Schuster Concrete	Annapolis Junction Plant, Laurel Plant, Monument Street Plant, Portable 18 Plant, Rockville Plant
Scioto Ready Mix	Alexandria Plant, Delaware Plant, Dublin Plant, New Albany Plant, Obetz Plant
Silvi Materials	Downingtown Plant, Englishtown Plant, Kingston Plant, Limerick Plant, Logan Plant, Morrisville Plant, Mt. Holly Plant, Philadelphia Plant, South Plainfield Plant, Southampton Plant
Stoneway Concrete	Black River Plant, Houser Plant, Seattle Plant
Tec-Crete Transit Mix Corporation	Jamaica Plant, Long Island City Plant
Thomas Concrete	Airport Plant, Alpharetta Plant, Atlanta Plant, Buckhead Plant, Charleston Plant, Charlotte Graham Street Plant, Doraville Plant, Greenville Plant, Morrisville Plant, Pooler Plant, Savannah East Plant, West Street Plant
Tilcon Connecticut Inc.	East Granby Concrete Plant, Hartford Concrete Plant, New Britain Concrete Plant, Norwich Concrete Plant, Old Saybrook Concrete Plant
Titan Virginia Ready-Mix	Centerville RM Plant, Clear Brook RM Plant, Dumfries RM Plant, Front Royal RM Plant, Leesburg RM Plant, Springfield RM (Plants 1 and 2), Stafford RM Plant, Sterling RM (Plants 1 and 2)
United Companies	Crested Butte Plant, Delta Plant, Gunnison Plant, Gypsum Plant, Minturn Plant, Montrose Plant, Powers Plant, Rifle Plant, River Road Plant, Steamboat Plant, Telluride Plant, Woody Creek Plant
US Concrete Mix / Smyrna Ready Mix	Bushwick Plant, College Point Plant, Jenna Plant, Long Island City Plant, Maspeth Plant, Mt. Vernon Plant, Smith Street Plant

**Fig. 2:**
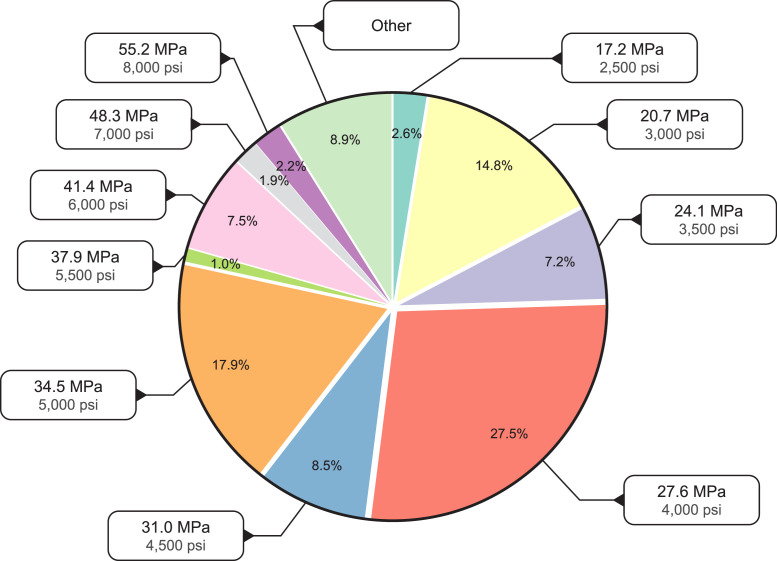
Breakdown of concrete compressive strength classes in the EPD dataset.

**Fig. 3:**
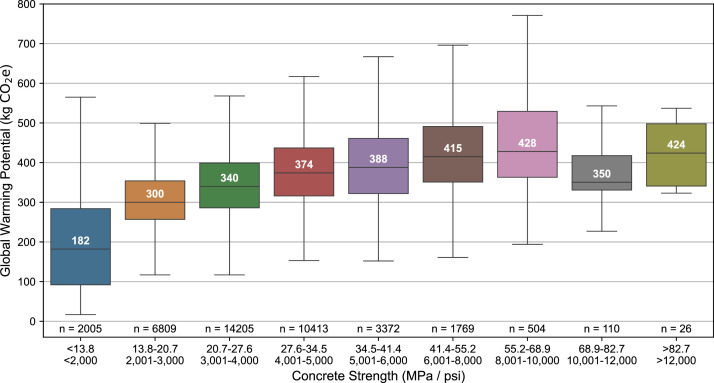
Concrete Compressive Strength and A1-A3 Global Warming Potential for all EPDs in the dataset. Note that this includes EPDs with different functional units (i.e., different curation times).

**Fig. 4:**
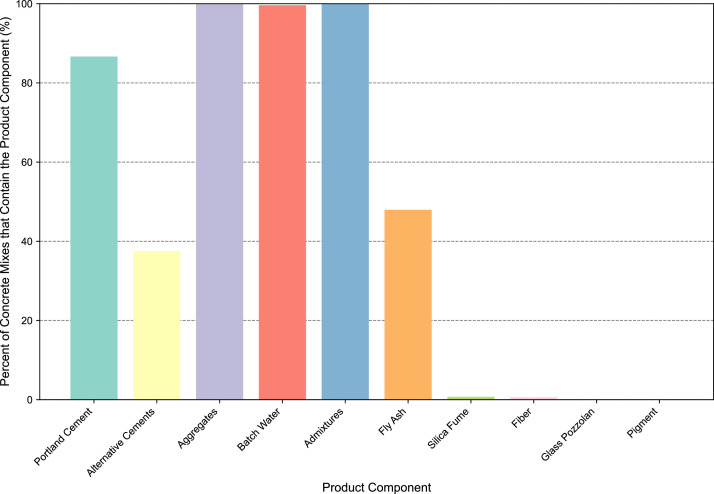
Breakdown of concrete mixture components. Note that the percentage of mixtures that contain any one product component is out of the entire dataset.

The dataset also represents a broad range of concrete compressive strength classes. Fig. 2 shows that though over half of the concrete mixtures have a design compressive strength between 27.6 MPa (4000 psi) to 34.5 MPa (5000 psi), several other strength classes are represented, including those below 17.2 MPa (2500 psi) and above 55.2 MPa (8000 psi). Because of the breadth of compressive strengths in the dataset, holistic assessments of strength against LCA mid-points, like Global Warming Potential, can be conducted, as demonstrated by Fig. 3. Additionally, the concrete mixtures also include up to 15 different product components: portland cement, alternative cements (slag cement, type 1 L cement, and hydraulic cement), aggregates (natural, crushed, and lightweight), batch water, admixtures (ASTM C494 and C260), fly ash, silica fume, fiber, glass pozzolan, and pigment. The percentage of concrete mixtures that include each component is shown in Fig. 4. Note that a single concrete mixture commonly includes several product components (for example, natural aggregate, crushed aggregate, portland cement, batch water, slag cement, and chemical admixtures).

Two strategies to efficiently parse and analyze the large dataset include the filter feature in Excel and reading the dataset in Python or an equivalent coding language. In Excel or Python, users can view or filter concrete mixtures from a specific concrete plant, geographical location, concrete compressive strength class, or LCA midpoint. Python can further filter the dataset to identify EPD information including material components and LCI sources. Doing so can enable sustainability researchers and building practitioners to analyze the environmental performance of different concrete mixtures currently available in the U.S.A. A study by Anderson and Moncaster [9] is an example of an analysis that can be done with this data.

## 3 Experimental Design, Materials and Methods

**Fig. 5:**
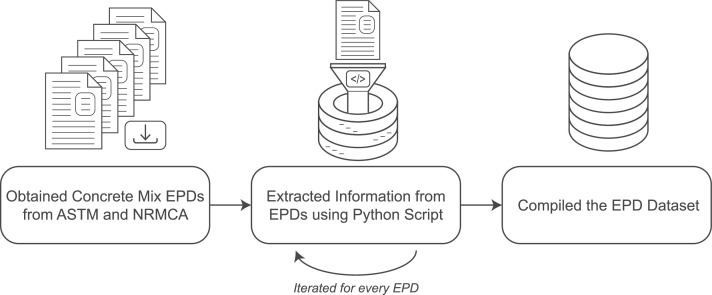
Method for generating the ready-mix concrete EPD dataset.

The information in the ready-mix concrete EPD database is obtained from EPDs made available online from ASTM [1] and NRMCA [2] between February and April of 2023. The internet links to obtain the EPDs are provided in the dataset column: “EPD Source Link.” The ready-mix concrete EPDs were downloaded as PDF files and put into subfolders based on the ready-mix concrete producer. The accompanying Python script (Concrete_EPD_Information_Mining_Python_Script.py) was written to extract the relevant information from each ready-mix concrete EPD automatically, circumventing the need for manually reading, interpreting, and reporting the information for each EPD. The Python script was run for every ready-mix concrete producer to obtain the compiled EPD dataset, as illustrated in Fig. 5. The Python script was created using Anaconda Navigator 2.4.1, using Jupyter Notebook with Python v. 3.8.

The Python script employed the open-access library pdf plumber [10] to extract text from each EPD .pdf file. The provided .py file can be employed on any Python environment with Python v. 3.0 or newer. The user can define the desired information for extraction (e.g., the LCA midpoints, ready-mix concrete plant locations), and the Python script then extracts the information from the read .pdf file and saves each entry. Once the for loop completes, the recorded data is written as a new .csv file which can then be assembled to the EPD dataset and cleaned to match the syntax and numerical format across all ready-mix concrete producers.

## Limitations

While the EPD database contains many entries, this is not an exhaustive list of concrete mixtures currently available. Second, the dataset includes ready-mix concrete produced only in the U.S.A.; no other countries are considered. Although no other countries are considered, a limitation with the analyzed EPDs is that European LCI audits are used for certain LCI items such as cleaning chemicals, indicating that the environmental performance for the items is similar within the U.S.A., but may contribute to the LCI variability and uncertainty. Third, the quality of the data and the amount of data available differs across concrete plant/ready-mix concrete producers. However, all EPDs are externally validated by a third-party organization. Lastly, EPDs have a five-year period that they are valid. Therefore, EPDs in the dataset may not be valid depending on the time that the future analysis is conducted. The earliest date that an EPD is not valid is April 3rd, 2024. Regarding the provided Python code, it should be noted that ready-mix concrete EPDs from different plants or manufacturers can differ from one another. Therefore, the provided code may need to be modified to correctly extract all information from an EPD.

## CRediT authorship contribution statement

**Jonathan Michael Broyles:** Conceptualization, Methodology, Software, Investigation, Data curation, Writing – original draft, Visualization. **Juan Pablo Gevaudan:** Methodology, Validation, Writing – review & editing. **Nathan Collin Brown:** Writing – review & editing, Supervision.

## Data Availability

Compiled Dataset of Concrete Mixture Environmental Product Declarations in the U.S.A. (Original data) (Mendeley Data)

## Appendix Supplementary materials

Image, application 1
